# Beyond Classification: A Case Report Exploring Paraphrenia in Late-Onset Psychosis

**DOI:** 10.1192/j.eurpsy.2025.2247

**Published:** 2025-08-26

**Authors:** M. O. Pires, M. Barroso, A. S. Pires, S. Mouta, I. F. Vaz, J. Nunes

**Affiliations:** 1 Unidade Local de Saúde da Guarda, Guarda, Portugal

## Abstract

**Introduction:**

Paraphrenia is a chronic psychotic disorder characterized by persistent delusions, with preservation of cognitive function and personality, which distinguishes it from schizophrenia, where cognitive decline is more pronounced. Although initially defined by Kraepelin, paraphrenia has been excluded from current diagnostic systems, complicating its distinction from schizophrenia and delusional disorders. Over time, experts like Mayer-Gross, Roth, and Munro have debated whether paraphrenia constitutes a distinct diagnostic entity, raising questions about its relevance in contemporary psychiatry.

**Objectives:**

To present a case that resembles paraphrenia and explore the clinical presentation and diagnostic process, particularly in the context of the disorder’s exclusion from diagnostic systems.

**Methods:**

This study is based on a case report, supported by a non-systematic review of relevant literature. Clinical data was collected throughout the patient’s treatment, and articles on paraphrenia were reviewed to provide historical and diagnostic context.

**Results:**

A 60-year-old woman, single, household cleaner, was brought by her sibling to the emergency department for presenting persecutory delusions, and auditory hallucinations in the prior four months. She believed her downstairs neighbors had installed cameras in her house, controlling her every move. She presented a depressed mood and difficulty falling asleep. Toxicology screening, blood work, and head-CT showed no changes. Due to treatment refractoriness, Clozapine was started. A formal neuropsychological assessment was carried out, which did not show cognitive deterioration. The patient revealed less preoccupation with delusions and hallucinations and was discharged two months after admission.

**Conclusions:**

In this case, the patient exhibited a chronic psychotic disorder marked by persecutory delusions and auditory hallucinations, without encapsulated delusional thinking. These symptoms had persisted for four months, during which the patient maintained personality stability and interpersonal functionality, displaying appropriate emotional responses and no cognitive decline. The onset occurred in middle age, with no psychiatric family history or evidence of premorbid issues. Significant stressors before symptom onset were identified as a likely trigger. This case highlights the persistence of psychotic symptoms with preserved cognitive and emotional stability, aligning with the diagnostic characteristics of paraphrenia. Despite exclusion from modern classification systems, the clinical presentation supports considering paraphrenia as distinct from other psychotic disorders, particularly in terms of its preserved functionality and absence of cognitive decline. This case underscores the need to continue the study on paraphrenia, and possibly rethinking its role in diagnostic frameworks, particularly in cases of late-onset psychosis.

**Disclosure of Interest:**

None Declared

